# The skeletal completeness of the Palaeozoic chondrichthyan fossil record

**DOI:** 10.1098/rsos.231451

**Published:** 2024-01-31

**Authors:** Lisa Schnetz, Richard J. Butler, Michael I. Coates, Ivan J. Sansom

**Affiliations:** ^1^ School of Geography, Earth and Environmental Sciences, University of Birmingham, Edgbaston, Birmingham B15 2TT, UK; ^2^ Department of Organismal Biology and Anatomy, University of Chicago, Chicago, IL 60637-1508, USA

**Keywords:** cartilaginous fishes, diversity, temporal pattern, sampling bias, environment

## Abstract

Chondrichthyes (sharks, rays, ratfish and their extinct relatives) originated and diversified in the Palaeozoic but are rarely preserved as articulated or partly articulated remains because of their predominantly cartilaginous endoskeletons. Consequently, their evolutionary history is perceived to be documented predominantly by isolated teeth, scales and fin spines. Here, we aim to capture and analyse the quality of the Palaeozoic chondrichthyan fossil record by using a variation of the skeletal completeness metric, which calculates how complete the skeletons of individuals are compared to estimates of their original entirety. Notably, chondrichthyan completeness is significantly lower than any published vertebrate group: low throughout the Silurian and Permian but peaking in the Devonian and Carboniferous. Scores increase to a range similar to pelycosaurs and parareptiles only when taxa identified solely from isolated teeth, scales and spines are excluded. We argue that environmental influences probably played an important role in chondrichthyan completeness. Sea level significantly negatively correlates with chondrichthyan completeness records and resembles patterns already evident in records of ichthyosaurs, plesiosaurs and sauropodomorphs. Such observed variations in completeness highlight the impact of different sampling biases on the chondrichthyan fossil record and the need to acknowledge these when inferring patterns of chondrichthyan macroevolution.

## 1. Introduction

Chondrichthyans (elasmobranchs and chimaeroids) are a highly successful class of predominantly predatory fishes. They originated and first diversified in the Palaeozoic and persist until the present day [1–3]. Extinct and extant chondrichthyans show adaptations to different habitats in both freshwater and marine environments, occupying various ecological niches (e.g. [4–8]). Chondrichthyan remains have been collected from all continents, highlighting their varied temporal and spatial distribution. Chondrichthyan skeletal conditions are recognized as highly derived [9,10], with key features including a microsquamous dermal skeleton and an endoskeleton almost entirely lacking bone, but with specialized mineralized cartilage encasing and, in places, replacing a largely unmineralized structure [11–14].

The early history of chondrichthyans has undergone major revision in recent years but is strongly influenced by the poor quality of their fossil record owing to the fragility of their skeletons (e.g. [10,15–17]). Evidence of chondrichthyans potentially dates to the Middle to Upper Ordovician, consisting of isolated scales of putative chondrichthyan origin [18–22]. Isolated teeth and articulated specimens of conventional (i.e. near-crown condition) chondrichthyan morphology are found from the Early Devonian onwards (e.g. [7,23–25]). The recognition of the acanthodians, a group of Palaeozoic spiny fusiform fishes, as a grade of the chondrichthyan stem-group, has partially filled stratigraphic gaps in the Silurian (e.g. [10,26–30]). These hypotheses of range extension have now been confirmed with body fossils and further diagnostic fragments from the Early Silurian of China [3,31,32]. However, how the quality of these various fossils and their differing degrees of completeness influence estimated patterns of diversity and phylogenetic relationship remains uncertain. Investigations into the diversity estimates of contemporary Palaeozoic fish groups such as actinopterygians have highlighted the taxonomic challenges and multiple biases affecting their fossil record but have not yet accounted for the effects of taphonomic biases [33,34]. Likewise, the totality of the early chondrichthyan dataset is rarely considered, and the nature and quality of the chondrichthyan fossil record, including acanthodians, has not been quantified to date.

While the fossil records of all groups are the fundamental sources of data for palaeontology, they are subject to inconsistent preservation on a variety of spatial, temporal, taxonomic and environmental scales (e.g. [35–39]). Biases resulting from geological, anthropogenic and taphonomic processes are commonly recognized to significantly influence observed patterns of diversity and fossil record quality (e.g. [40–44]). The influence of sampling and collector biases on our understanding of macroevolutionary changes and patterns of diversity through time has been highlighted in recent years (e.g. [45–51]). A common approach to addressing fossil record quality involves estimating fossil specimen quality by assessing levels of fossil specimen completeness. The most comprehensive approach was developed by Mannion & Upchurch [52] and presents completeness metrics that precisely quantify the completeness of individual specimens and species. Initially applied to sauropodomorph dinosaurs, subsequent studies have used these metrics to quantify fossil record completeness for several terrestrial [53–64] and marine vertebrate groups [65–67], including acanthodian stem-chondrichthyans [68].

Here, we use modified versions of the previously published skeletal completeness metrics [52,69,70] to quantitatively examine the quality of the Palaeozoic chondrichthyan fossil record, to our knowledge for the first time. We expand the preliminary assessment of the stem-chondrichthyan acanthodian completeness [68] to the non-acanthodian, and consequently total-group, chondrichthyans from the Palaeozoic. We correlate variations in completeness through geological time with potential palaeobiological and palaeoecological variables, including taxonomic richness, changes in sea level and depositional environment. We identify potential biases and highlight gaps that might affect the chondrichthyan fossil record by statistically comparing completeness values between different taxonomic groups, geographical regions and depositional environments. These results are likely to guide future endeavours in resolving fossil record quality biased by missing data and add to our knowledge of factors shaping our perspectives on early chondrichthyan evolution, and thus early gnathostomes in general.

## 2. Material and methods

### 2.1. Completeness metrics

The two most commonly used metrics for estimating the completeness of a fossil vertebrate skeleton are the character completeness metric (CCM) and the skeletal completeness metric (SCM) of Mannion & Upchurch [52]. Previous studies have shown a significant positive correlation between CCM and SCM in different vertebrate groups, suggesting that the metrics detect similar signals in fossil record quality [52,66]. Details regarding the potentially problematic usage of CCM for groups with a predominantly cartilaginous skeletons are detailed by Schnetz *et al*. [68]. We follow Cashmore & Butler [61] and Schnetz *et al*. [68] in assessing the quality of the chondrichthyan fossil record using SCM rather than CCM in this study. SCM was established with two variants: SCM1, which establishes the completeness of the most complete specimen for a given species or taxon, and SCM2, which estimates completeness using all specimens of a given species or taxon (a composite of all investigated specimens). The latter variant has been preferred in subsequent studies [53,56,58,61,63,68], and is also used here.

**Figure 1.**
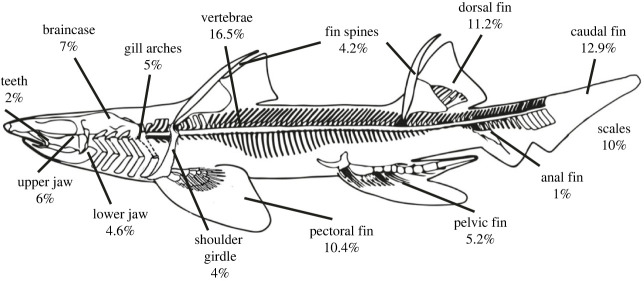
Skeletal reconstruction of *Tristychius arcuatus* Agassiz, 1837 (modified from [71]), illustrating the mean skeletal proportions of chondrichthyan body exemplar 1.

We used a combination of two completeness metrics, the Mannion & Upchurch [52] and the Beardmore *et al*. [69,70] metrics, to calculate Palaeozoic chondrichthyan completeness (figure 1), following the approach detailed by Schnetz *et al*. [68] on the stem-chondrichthyan acanthodian grade. Detailed information on the scoring system and descriptions for each individual skeletal region is accessible in the electronic supplementary material. The majority of chondrichthyan specimens are preserved in two-dimensional lateral compression. To account for this, we report completeness based on the side visible (assuming a similar preservation of the other side), following previous completeness studies [60,61,65,68].

### 2.2. Chondrichthyan completeness data

In this study, we build upon the preliminary assessment of stem-chondrichthyan acanthodian grade completeness [68] and assess the completeness of the non-acanthodian stem-chondrichthyans and crown group chondrichthyans throughout the Palaeozoic. Therefore, we initially exclude the acanthodian grade *sensu* Dearden *et al*. [29] and Frey *et al*. [17] for the analyses of chondrichthyan completeness. We include the stem-group chondrichthyan taxa that are more closely related to the chondrichthyan crown (e.g. *Pucapampella*, *Doliodus* and *Gladbachus*) to compare and contrast patterns with the previously published acanthodian grade, and arbitrarily define chondrichthyans as *Gladbachus* and all closer relatives of living chondrichthyans (the conventional chondrichthyans *sensu* [17]). The Silurian stem-chondrichthyan *Shenacanthus* [31] was unavailable for inclusion at time of analysis, and we recognize that its estimated relationship to crown chondrichthyans is tenuous. We include the Ordovician shark-like scale taxa *Tantalepis*, *Solinalepis*, *Canyonlepis* and *Tezakia* which are currently assigned to the Chondrichthyes *sensu* *lato* [20–22]. We also include Silurian scale- and fin spine-based taxa such as elegestolepids [72,73], mongolepids [22,74] and sinacanthids [75,76], some of which might branch from within the acanthodian grade, but differ in a number of significant features (notably histology and scale growth) with the acanthodians as currently defined [30]. Additionally, these taxa are overlooked in phylogenetic analyses of the total-group chondrichthyans [77].

We compiled a dataset of 3021 specimens of Palaeozoic chondrichthyans, which is up to date as of August 2021 (see the electronic supplementary material for a full list of included genera and species). The final dataset contains 800 species and 287 genera of Palaeozoic chondrichthyans. Primary information was gathered from first hand observation of specimens during museum visits and augmented with information from the literature (both illustrations and text). Information on the lithostratigraphy (e.g. geological formation), geographical locality and chronostratigraphic age were recorded for each specimen together with their completeness scores. Additionally, palaeoenvironmental information was gathered from published sources where detailed sedimentological descriptions for specimen occurrences were available. Taxonomic information from museum catalogues was checked for validity and corrected to the latest accepted taxonomic name and systematic position if applicable. Taxa were included based on the most recent taxonomic literature and reviews (e.g. [4,7,78]).

The initial data were divided into two subsets: one excluding isolated tooth-based taxa and one excluding isolated tooth, scale and fin spine-based taxa. Tooth-based taxa comprise 65%, fin spine-based taxa 12% and scale-based taxa 6% of total species. Some of the analyses were run multiple times using each of these subsets. Lastly, we added the acanthodian data from Schnetz *et al*. [68] to our non-acanthodian stem-chondrichthyan and crown group chondrichthyan data for selected analyses to compare total-group chondrichthyan completeness patterns throughout the Palaeozoic with either dataset.

### 2.3. Time series

Temporal analyses and time series were conducted using stage-level time bins, starting from the Darriwilian (Middle Ordovician) up until the Changhsingian (Lopingian, Upper Permian). Chronological ages, time bin lengths and stage midpoints were established in accordance with the latest information from the International Commission on Stratigraphy stratigraphic charts (v2020/03; [79]). The resolution for the Pridoli (Upper Silurian) had to be limited to an epoch-level time bin as there are currently no officially recognized stages within this epoch. We follow previous analyses in providing a stage-level resolution for completeness (e.g. [61,63,68]), which is also the norm for most macroevolutionary and macroecological studies of the fossil record through deep time. Mean and median SCM2 completeness scores for each time bin were calculated using all taxa occurring within that time bin. Sampled in-bin occurrences of specimens were used to determine the temporal range of individual taxa.

### 2.4. Taxonomic groups

To compare if completeness scores differ significantly within Palaeozoic Chondrichthyes, we calculated skeletal completeness for total-group Holocephali (which include the symmoriiforms) and total-group Elasmobranchii following the most recent phylogenetic analyses by Coates *et al*. [10], Dearden *et al*. [29] and Frey *et al*. [17] (details of the iterative tests and updates of the datasets can be found in the electronic supplementary material). We contrasted these tree-based systematics by using traditional taxonomic schemes based on dental morphologies employed by Ginter *et al*. [7] and in part by Stahl *et al*. [78], grouping chondrichthyan completeness into the subclasses Elasmobranchii (which include the symmoriiforms) and Euchondrocephali. This allowed us to test if SCM2 distributions of subgroups are robust under conflicting estimates of inter-relationships. Additionally, chondrichthyans were grouped into the following orders to assess differing levels of completeness on a finer scale: Altholepidiformes, Antarctilamniformes, Bransonelliformes, Chimaeriformes, Chondrenchelyiformes, Cochliodontiformes, Copodontiformes, Coronodontida, Ctenacanthiformes, Elegestolepidida, Eugeneodontiformes, Helodontiformes, Hybodontiformes, Iniopterygia, Menaspiformes, Mongolepidida, Omalodontiformes, Orodontiformes, Petalodontiformes, Phoebodontiformes, Polymerolepidiformes, Psammodontiformes, Sinacanthida, Symmoriiformes, Synechodontiformes and Xenacanthiformes. See the electronic supplementary material for details on which taxa were assigned to which order and supporting references were applicable. Taxa were included based on the most recent taxonomic literature and reviews (e.g. [4,7,78]).

### 2.5. Spatial correlations

Completeness scores were grouped by the present-day hemispheres and geographical regions in which they were collected, to assess if the chondrichthyan fossil record quality varies on a global scale. Palaeozoic chondrichthyans have been recovered from all modern-day continental regions, including taxa from Africa (18), Asia (78), Australia and Oceania (6), Antarctica (5), North America (456), South America (14) and Europe (297). The influence of Lagerstätten (sites of exceptional fossil preservation) can lead to biases in the overall trend of completeness of any given group through time (see previous completeness studies: [53,56,60,61,63]). However, clearly defined Lagerstätten containing Palaeozoic chondrichthyans are limited to a few localities (e.g. the Bear Gulch and Mazon Creek biotas) and are unlikely to significantly bias the overall completeness patterns. We therefore follow previously published completeness studies on other marine vertebrates [65–68] and do not separate taxa derived from concentration or conservation Lagerstätten for statistical comparisons.

### 2.6. Environment

We classified information on the depositional settings of each specimen into benthic assemblage zones (BAs) to understand whether levels of completeness are influenced by the taphonomic, preservational and environmental settings they were deposited within. BAs are categorized into fresh water (BA0); intertidal above typical wave base (BA1); shallow subtidal and/or lagoon (BA2); deeper subtidal and/or reefs (BA3); middle to outer shelf (BA4 and BA5) and shelf margin towards the bathyal region (BA6) [80–82]. While there is some inconsistency about the exact distinction between BA4 and BA5, we here consider BA4 to be the limit of subtidal influence before getting into the deepest extremities of the shelf in BA5. Completeness scores for each taxon were subdivided between all BAs they were deposited in. Additionally, completeness of taxa was classified as originating from either freshwater or marine settings. Changes in average sea level through time were furthermore used as a sampling proxy for the environmental effect on chondrichthyan completeness. Average sea level data was derived from Hannisdal & Peters [83] who provided a composite Phanerozoic sea level reconstruction based on previous studies. We excluded the value for the upper Wuchiapingian in the environmental analyses, which also represents the last sea level value for Palaeozoic data. We argue that this point represents an outlier which skews the correlations and distorts the patterns between sea level and completeness. There are very limited sea level values for the middle and upper Permian stages (a total of two) in the reconstructions and there is a considerable drop in sea level between the two values. Data for the preceding and subsequent stages are missing and thus, this one value represents an extreme point compared to the rest of the Palaeozoic data.

### 2.7. Statistical analysis

All statistical analyses were performed in R v. 4.2.0 [84]. We largely follow the statistical protocols used in the most recent completeness studies (e.g. [60,61,63,68]). Time-series plots were generated using the package ggplot2 [85] and non-temporal completeness distribution plots were generated using both ggplot2 and the package vioplot [86]. Linear regressions were used to test series of completeness trends through time. Generalized least-squares regressions (GLS) were used to test completeness series trends through time, implemented with a first-order autoregressive model (corARMA) to reduce the chance of overestimating the statistical significance of the regression lines owing to temporal autocorrelation. GLS were calculated using the function gls() in the R package nlme [87]. Time series were log-transformed prior to analysis, ensuring normality and homoskedasticity of residuals. Likelihood-ratio-based pseudo-R^2^ values were calculated to determine the amount of variance explained by the GLS models using the function r.squaredLR() in the R package MuMIn [88].

GLS autoregressive models were used to make time-series comparisons between completeness metrics through time as well as compare temporal changes in completeness to multiple combinations of potential explanatory variables (species richness, time bin length, stage midpoints and sea level). Stage midpoints were used to test for a general trend through time and time bin lengths were used to examine any effects of variable time durations of the different stages. We used 'not available' (NA) for stages that were not represented in the sea level reconstructions by Hannisdal & Peters [83] in order to calculate our regression analyses. To measure model fit of the data together with model complexity, Akaike's information criterion (AICc) and Akaike weights were calculated using the functions AICc() of the R package qpcR [89] and aic.w() of the R package phytools [90].

Non-parametric Mann–Whitney–Wilcoxon tests were used to perform non-temporal pairwise comparisons of completeness values by assessing differences in standard deviations and medians of datasets. For comparisons of more than two datasets/subsets, Kruskal–Wallis tests were calculated to determine any dominances of variables in the dataset. The chondrichthyan completeness values were additionally compared to the published SCM2 data of other vertebrate groups, including sauropodomorphs ([52]; updated dataset by Cashmore *et al*. [63]), pelycosaurs [55], ichthyosaurs [65], parareptiles [58], plesiosaurs [66], bats [60], theropods [61] and acanthodians [68]. These groups constitute the only available published comparators for skeletal completeness to date.

## 3. Results

### 3.1. Chondrichthyan completeness through time

**Figure 2.**
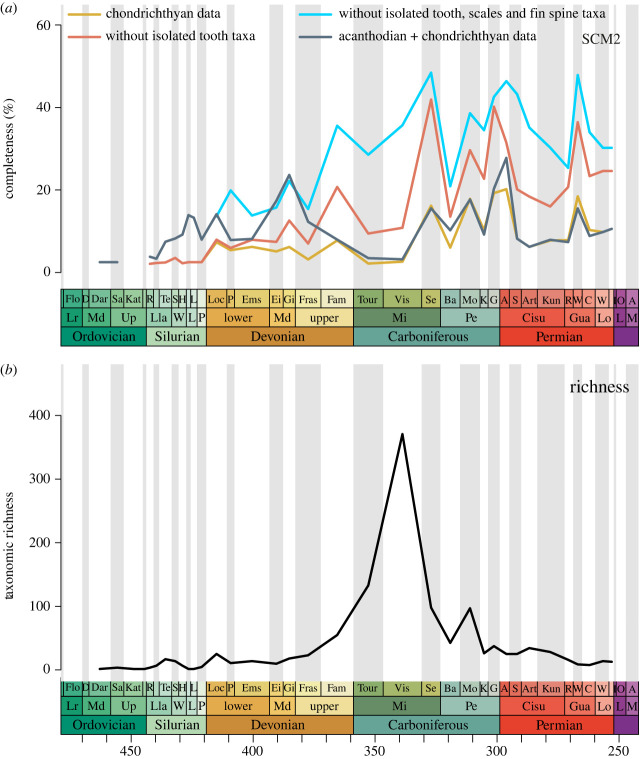
Changes in Palaeozoic chondrichthyan completeness and raw taxonomic richness through time. (*a*) Mean skeletal completeness and (*b*) raw taxonomic richness (species level).

Mean completeness is initially low within the Middle–Upper Ordovician and Silurian (2.1–3.5%), followed by slight increases through the Devonian (approx. 3.2–7.8%) (figure 2*a*). Completeness dips initially in the early Carboniferous but peaks in the Serpukhovian (16.2%), Moscovian (17.9%) and again in the Gzhelian–Asselian (19.2–20.2%) at the Carboniferous–Permian boundary. The remainder of the Permian exhibits lower levels of completeness except for a peak in the Wordian (18.5%) and a slight increase in completeness towards the end of the Permian. Excluding isolated tooth-based chondrichthyan taxa results in higher mean SCM2 values throughout most of the Palaeozoic time bins. SCM2 values do not change considerably in the Ordovician, Silurian and Lower–Middle Devonian but are overall 7–26% higher than the original dataset from the Upper Devonian onwards. The general patterns and peaks are retained except for a pronounced peak in the Gzhelian (40.2%), rather than in both the Gzhelian and Asselian as in the original dataset. Removal of isolated tooth-, isolated scale- and isolated fin spine-based taxa yields the overall highest mean chondrichthyan skeletal completeness values of any subset throughout the Palaeozoic, ranging between 10 and 48%. In this subset, there are no values for the Ordovician and Silurian, except for the Sheinwoodian.

Median chondrichthyan SCM2 initially depicts similarly low values to mean SCM2 that range between 0.5% and 2.5% throughout the stages of the Palaeozoic (electronic supplementary material, figure S1). No peaks or lows are identified. Excluding isolated tooth-based taxa from the chondrichthyan dataset alone greatly changes the median SCM2 completeness pattern for chondrichthyans through the Palaeozoic (electronic supplementary material, figure S1). While completeness stays low throughout most stratigraphic stages, notable peaks are recovered in the Givetian (9.2%), Serpukhovian (32.7%), Moscovian (13.4%), Gzhelian (39.1%), Wordian (34%) and Capitanian (30.6%). Values remain higher in the uppermost stages of the Permian compared to the previous periods. Removal of isolated tooth-, isolated scale- and isolated fin spine-based taxa yields the overall highest median skeletal completeness values (electronic supplementary material, figure S1). As observed in the mean completeness patterns, there are no data for the Ordovician and Silurian with the exception of the Sheinwoodian. Similar, albeit somewhat higher, peaks to the isolated tooth-based subset are recovered, with the addition of a pronounced peak in the Pragian (19.9%) and Asselian (44.9%).

**Figure 3.**
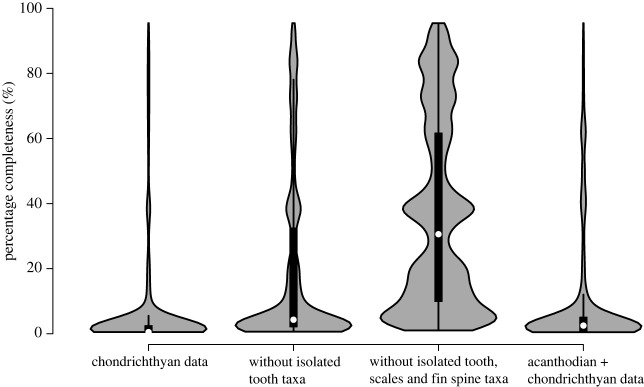
Distribution of original chondrichthyan SCM2 values compared to the subsets.

The distributions of the total SCM2 values are significantly different from the two subsets (excluding isolated tooth-based taxa as well as excluding all isolated tooth, scale and fin spine-based taxa) (electronic supplementary material, table S1; figure 3). Time-series comparisons show significant positive relationships between the original SCM2 values and SCM2 of all subsets (electronic supplementary material, table S2). Additional GLS comparisons through time reveal a significant negative correlation between original chondrichthyan SCM2 and sea level (*p* = 0.0086, *R*^2^ = 0.80) (electronic supplementary material, table S3). Higher skeletal completeness is found in time bins with lower sea level. SCM2 time series are best explained by the three GLS models including sea level (model 4), including stage midpoints + sea level (model 9) and including stage midpoints + richness + sea level (model 10) (electronic supplementary material, table S3). All three models have high strength (*R*^2^ values ranging between 0.80 and 0.85), with the sea level coefficient in model 4 being significant (*p* = 0.0086) and the stage midpoint coefficient in model 9 (*p* = 0.0255) and 10 (*p* = 0.05) being significant.

### 3.2. Correlations with taxonomic richness

Chondrichthyan raw taxonomic richness (species level) is low in the Ordovician and Silurian apart from a small peak in the Telychian (species count = 16) (figure 2*b*). Richness peaks again in the Lower Devonian Lochkovian (species count = 24) but remains stable and at low levels until the Upper Devonian. It then rises steeply from the Famennian onwards before reaching a climax in the Visean stage of the Carboniferous (species count = 361). Raw taxonomic richness subsequently falls rapidly in the Serpukhovian and Bashkirian before increasing once more with a peak in the Moscovian (species count = 94). From the end-Carboniferous onwards, richness remains at lower levels and slightly decreases throughout the Guadalupian but remains fairly stable in the Lopingian towards the Permian–Triassic boundary. GLS models show that a combination of time bin length, SCM2 and sea level provides the best explanation for raw chondrichthyan taxonomic richness (model 13) (electronic supplementary material, table S4). The relationship between taxonomic richness and time bin length is significant and positive (*p* = 0.012; *R*^2^ = 0.75) but none of the other explanatory variables have a significant relationship with richness. The models that best explain taxonomic richness, ranked by AIC weight values, include time bin length + sea level (model 9), time bin length + SCM2 + sea level (model 13) and stage midpoints + SCM2 + time bin length + sea level (model 15), but only the time bin length coefficient is significant in the three models.

### 3.3. Taxonomic groups

Elasmobranchii and Holocephali share similar SCM2 distribution shapes, with low median completeness values (1.05 and 0.5%, respectively) (electronic supplementary material, figure S2). Taxonomic grouping into Elasmobranchii and Euchondrocephali results in similar bottom-heavy SCM2 distributions, with identical median values to the phylogenetic divisions (1.05 and 0.5%). However, the distributions are found to be significantly different (Elasmobranchii–Holocephali: *W* = 57001.5, *p* = 6.48 × 10^−14^, Elasmobranchii–Euchondrocephali: *W* = 78703.5, *p* = 1.68 × 10^−26^). When chondrichthyans are further divided into orders, Iniopterygia have the highest median SCM2 value (68.3%) of any subgroup and have a markedly different distribution to any of the other taxonomic groups (electronic supplementary material, figure S3). Following this group, Antarctilamniformes (14.3%) have the next highest median SCM2 distribution. The remaining subgroups all have median SCM2 values of less than 10% and most of the SCM2 scores are concentrated at around 0–10%. A Kruskal–Wallis test suggests that the variance of completeness distributions is dominated by one or more subgroups (*H* = 346.7, *p* *<* 2.2 × 10^−16^). Results of Mann–Whitney–Wilcoxon tests between each subgroup are provided in the electronic supplementary material, table S5. Iniopterygians are found to have significantly different SCM2 scores to most subgroups.

### 3.4. Comparisons with the completeness of other vertebrate groups

**Figure 4.**
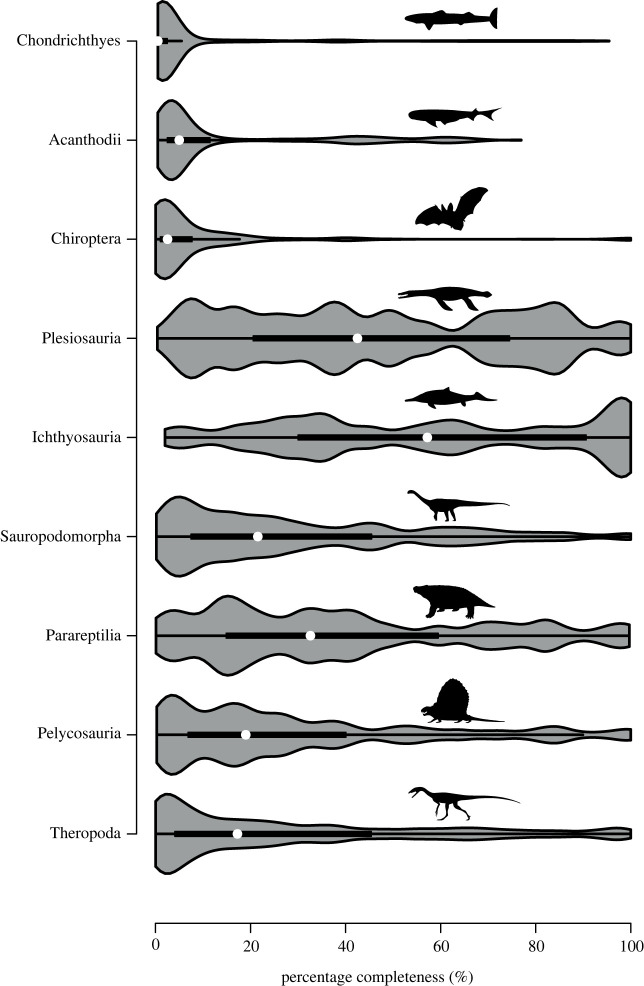
Range of chondrichthyan SCM2 values compared to other vertebrate groups. Comparative values from bats [60], plesiosaurs [66], ichthyosaurs [65], sauropodomorphs [52,63], parareptiles [58], pelycosaurs [55], theropods [61] and acanthodians [68]. Silhouettes taken from http://phylopic.org/ and include work by N. Tamura, G. Monger, S. Hartman, D. Bogdanov, Karkemish and Smokeybjb.

Chondrichthyan completeness values were plotted against SCM2 values of other published vertebrate groups to facilitate comparisons (figure 4). These previously published completeness distributions are limited to aquatic and non-aquatic tetrapods aside from a previously published acanthodian completeness [68]. The distribution of chondrichthyan median SCM2 scores, visualized by violin plots, is significantly lower than any tetrapod group investigated, including the bat fossil record (electronic supplementary material, table S6). Similar to bats and acanthodians, completeness of Palaeozoic chondrichthyans exhibits a distribution of most SCM2 values at low percentages with low numbers of highly complete taxa. A subset excluding isolated tooth-based taxa yields higher median completeness values than found in bats but is still considerably lower than other tetrapod groups (electronic supplementary material, figure S3 and table S7). Once isolated teeth, fin spine and scale-based taxa are removed from the chondrichthyan dataset, SCM2 distribution and median value change drastically. There is no statistically significant difference between chondrichthyan SCM2 and that of either parareptiles or pelycosaurs (electronic supplementary material, table S8). Median chondrichthyan SCM2 values are significantly higher than that of both sauropodomorph and theropod dinosaurs upon removal of these isolated remains from the dataset. Plesiosaur and ichthyosaur completeness both remain considerably higher than chondrichthyan completeness.

### 3.5. Acanthodian versus non-acanthodian chondrichthyan completeness

Comparing non-acanthodian chondrichthyans to the previously investigated acanthodian group [68], SCM2 of both groups are significantly different and acanthodians have a slightly higher median distribution while chondrichthyans have a greater range of completeness percentage values. When both groups are combined to represent the chondrichthyan total-group, the distribution of SCM2 scores is flattened but the median is slightly increased relative to the original chondrichthyan dataset (electronic supplementary material, figure S4b).

Total-group chondrichthyan SCM2 distribution values are statistically significantly different from the non-acanthodian chondrichthyan SCM2 values (electronic supplementary material, table S1; figure 4).

Addition of the acanthodian data to the chondrichthyan dataset for time-series comparisons leads to an increase in mean SCM2 values by 5–10% through most of the Silurian with a notable peak in the Gorstian–Ludfordian (13.9–13.3%) compared to the non-acanthodian chondrichthyan data alone (figure 2). SCM2 is higher throughout most of the Devonian (until the Famennian) as well. The peaks in the Lochkovian (14.1%) and Givetian (23.6%) especially show higher levels of completeness. Completeness does not change significantly post-Devonian apart from an increased peak in the Asselian (27.8%). Median SCM2 values of the total-group chondrichthyans, however, do not significantly increase throughout the Palaeozoic compared with the non-acanthodian chondrichthyan data (electronic supplementary material, figure S1). Completeness does peak slightly in the Gorstian–Ludfordian (both 8.4%) and plateaus on moderately higher values from the Pragian through to the Frasnian (around 4.3 and 5.1%), with two more peaks later on in the Moscovian (4.7%) and Asselian (8.1%).

### 3.6. Geographical completeness

**Figure 5.**
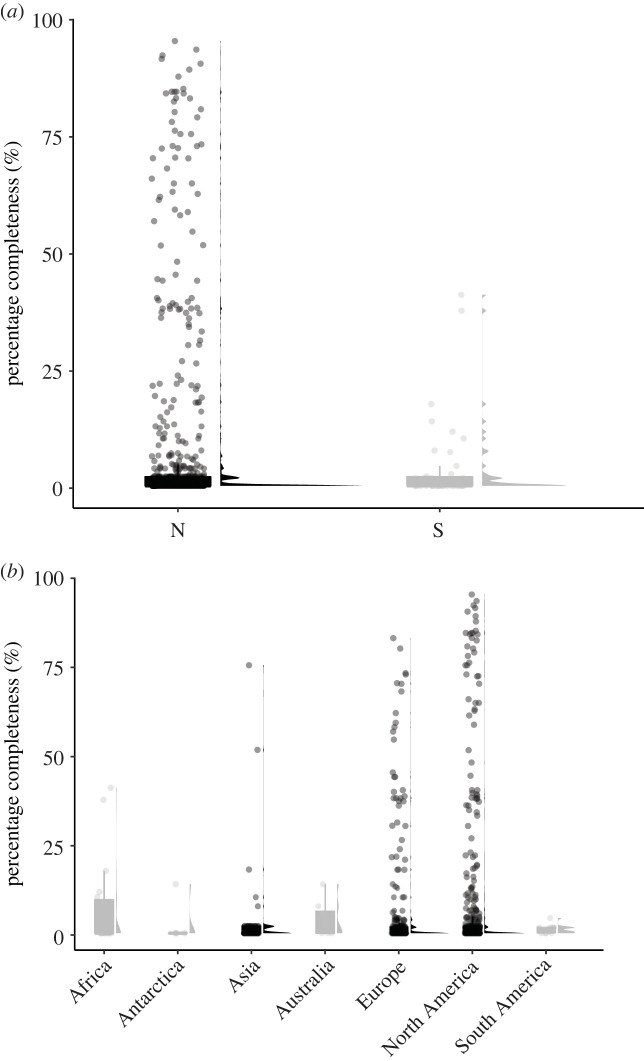
Distribution of chondrichthyan SCM2 scores between the modern Northern and Southern Hemispheres (*a*), and between the different continents (*b*).

Chondrichthyan species from Northern Hemisphere localities are not significantly more complete than those from the Southern Hemisphere when compared using Mann–Whitney–Wilcoxon tests (*W* = 15491, *p* = 0.28) and have similar distribution patterns with most SCM2 scores concentrated at low percentages (figure 5*a*). However, the range of SCM2 values is greater in taxa from the Northern Hemisphere (0.5–95.5%) whereas the Southern Hemisphere does not yield completeness values above 41.3%.

When divided into continental regions, Europe, North America and Asia all share similar distributions of the majority of completeness values but their range of percentage scores reaches to well above 70–80% (figure 5*b*). South America exhibits the smallest range of SCM2 distribution with a minimum of 0.5% and a maximum of 4.8%. The SCM2 distributions of Australia and Oceania and Antarctica are very similar in shape and median values. Africa has a moderately high range of completeness values, reaching maximum values of 41.3% but only 0.5% median completeness. The chondrichthyan SCM2 distributions recovered from each continent are all statistically similar to one another (electronic supplementary material, table S10). Additionally, Kruskal–Wallis tests do not indicate a strong dominance of any of the continents (*H* = 3.2664, *p* = 0.77).

### 3.7. Sea level correlations

**Figure 6.**
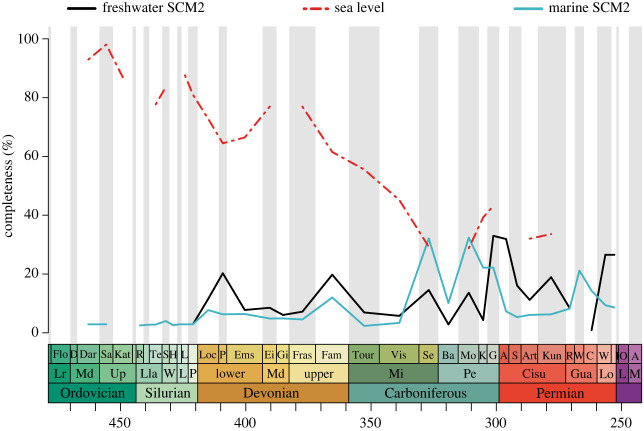
Chondrichthyan completeness through time based on freshwater versus marine occurrences plotted against sea level changes. Sea level values represent arbitrary values taken from Hannisdal & Peters [83].

There is a significant negative correlation between chondrichthyan completeness and changes in average sea level through time: chondrichthyan SCM2 values are higher during times of lower sea level and lower when sea level rises (figure 6 and table 1). When chondrichthyan SCM2 values are grouped into marine and freshwater completeness, there is a significant negative relationship between marine SCM2 and sea level, whereas freshwater SCM2 does not significantly correlate with sea level. Upon removal of the isolated tooth-based taxa from the dataset, average sea level is still significantly correlated with marine and total SCM2 values (electronic supplementary material, table S11). When the acanthodian SCM2 values from the previous investigation are added to the chondrichthyan dataset, a slightly positive and significant correlation between freshwater and sea level is recovered which stands in contrast with the other analyses (electronic supplementary material, table S12).

**Table 1.** Results of pairwise comparisons for sea level trends with chondrichthyan completeness using GLS. (Statistically significant results indicated in italics.)

comparison	slope	*t*-value	*p*-value	*R*^2^
total SCM2∼sea level	−1.3	−5.1	*0.0001*	0.87
marine SCM2∼sea level	−1.6	−4.4	*0.0003*	0.85
freshwater SCM2∼sea level	−0.24	−0.75	0.47	0.92

### 3.8. Depositional and environmental variation

SCM2 values recovered from chondrichthyans deposited in freshwater environments are significantly different to those in marine settings (*W* = 509.5, *p* = 0.03), but median SCM2 values are both low (0.5%) and neither setting demonstrates higher overall SCM2 completeness. Freshwater SCM2 values have a slightly higher upper interquartile range than marine SCM2 values which in turn have the highest maximum percentages (electronic supplementary material, figure S5). Plotting the values through time, freshwater SCM2 values are higher throughout the Devonian and the Tournaisian-Visean in the Carboniferous as well as most of the Permian (figure 6). Marine SCM2 peaks in the Serpukhovian and throughout most of the Pennsylvanian in the Carboniferous. The first chondrichthyan occurrences in the Ordovician and Silurian are from strictly marine deposits but marine SCM2 then remains lower than freshwater SCM2 until the late Mississippian.

When the environmental information is categorized into individual BAs for more detail, a Kruskal–Wallis test suggests that the variance of SCM2 distributions between the different BAs is strongly dominated by one or more of them (*H* = 36.451, *p* = 2.25 × 10^−6^). Mann–Whitney Wilcoxon tests further indicate a significant difference for SCM2 from BA0 compared to BA2, BA3 and BA4, and BA1 compared to BA4. Median SCM2 values recovered from BA2 BA3 and BA4 are all significantly different to BA5 and BA6 but not to each other (table 2). Further GLS pairwise analyses reveal significant relationships between each BA1, BA2, BA3, BA5 and BA6 and total SCM2 through time (electronic supplementary material, table S13). BA0 and BA4 do not show significant relationships with total SCM2 through time. Violin plots show similar distributions of SCM2 scores for all BAs (electronic supplementary material, figure S6).

**Table 2.** Results of pairwise comparisons between BA and total chondrichthyan SCM2 using GLS. (Statistically significant results indicated in italics.)

comparison	slope	*t*-value	*p*-value	*R*^2^
BA0 SCM2∼total SCM2	0.62	2.0	0.058	0.18
BA1 SCM2∼total SCM2	0.75	6.4	*< 0.0001*	0.89
BA2 SCM2∼total SCM2	0.53	3.1	*0.0041*	0.26
BA3 SCM2∼total SCM2	0.53	2.2	*0.038*	0.34
BA4 SCM2∼total SCM2	0.45	1.5	0.14	0.31
BA5 SCM2∼total SCM2	1.0	4.6	*0.0002*	0.63
BA6 SCM2∼total SCM2	1.3	3.9	*0.0009*	0.48

**Figure 7.**
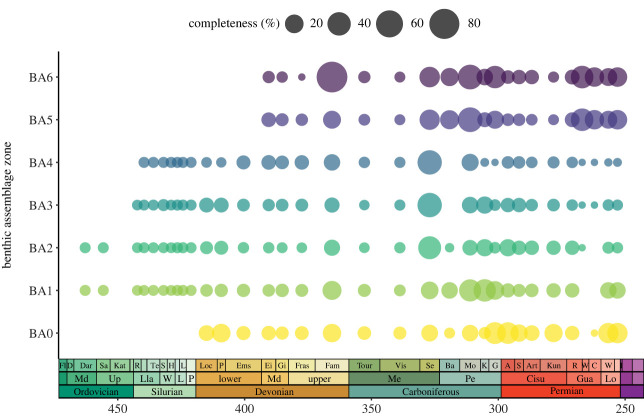
Changes in chondrichthyan completeness through time based on benthic assemblage (BA) zones.

Mean SCM2 through time sorted by individual BAs shows that completeness is initially restricted to low levels in BA1 and BA2 in the Ordovician before extending to BA1–4 in the Silurian (figure 7). Time series of mean SCM2 patterns of taxa deposited in BA1 and B2 closely resemble each other throughout most of the Palaeozoic. Similarly, completeness values for zones BA3 and BA4 follow a resembling pattern of mostly low levels throughout the Palaeozoic, and prominently, have no values for the Bashkirian in the Carboniferous. Mean SCM2 values of chondrichthyan taxa deposited in BA0 are restricted to the Devonian and upwards, with highest numbers in the Gzhelian–Asselian, Kungurian and Wuchiapingian–Changhsingian. Chondrichthyan SCM2 values from the deeper water BA5 and BA6 zones are limited to the Middle Devonian and upwards, with low scores initially in the Eifelian to Frasnian before heavily increasing in the Famennian (84.7% BA6). BA5 and BA6 completeness show similarly high patterns throughout most of the Carboniferous except for a low in the Tournaisian-Visean (2–4% SCM2) of the Mississippian. The patterns diverge slightly in the early Permian with higher completeness values reported for BA6 occurrences but merge again in the Guadalupian and Lopingian to show uniformly high scores.

## 4. Discussion

The chondrichthyan fossil record through time is dominated by sampling of isolated skeletal remains, namely teeth, scales and fin spines. Thus, it is not surprising that in comparison to total SCM2, the two subsets excluding taxa based on isolated skeletal material (teeth only and all teeth, scales and fin spines) yield overall significantly higher mean completeness values through time (figures 2 and 3). This shows how intensely specific isolated remains influence the observed completeness patterns of Palaeozoic chondrichthyans. Throughout the geological history of chondrichthyans, isolated teeth, and, to a lesser extent, isolated scales and fin spines, have constituted most of the available material employed to build a classification of this clade at ordinal and lower taxonomic levels (for an extensive summary, see [7,91]). This practice has been widely accepted for isolated tooth material, but a consistent application of a taxonomic standard for naming and assigning chondrichthyan taxa on the basis of isolated scales and fin spines has yet to be established. While taxonomic systems based on isolated scales have been developed for taphonomically similar groups or grades such as thelodonts and stem-chondrichthyans [77,92,93]), they have not been extended to the total-group chondrichthyans. However, the extent to which information contained in isolated scales can be used to infer characters for taxonomic and phylogenetic placement is debated and the limitations have been highlighted previously [68,94]. In the absence of such a concept, there is great potential for taxonomic ‘oversplitting' (e.g. *Akmonistion zangerli* includes *Lambdodus hamulus*, *Cladodus pattersoni*, *Cladodus exilis*, *Stemmatias simplex* and denticles cf. *Petrodus*: [95]) or ‘over-lumping' at species and genus levels. Indeed, such a tendency of taxonomic 'over-splitting' of poor-quality material has been reported before and linked to the inability to recognize characters that link specimens [55].

The predominantly cartilaginous composition of chondrichthyan skeletons limits the extent of fossil material to work with as cartilage, even with calcified tesserae, fossilizes poorly and only under favourable depositional conditions. While palaeontologists are extracting more information from previously inaccessible fossils (e.g. from concretions) following technological advances such as computed tomography scanning, a preservational bias towards isolated remains is still largely at play (e.g. [17,29,96–98]). Of these mineralized fragments, isolated teeth consist of layers of dentine and enameloid which are some of the hardest biological tissues, often resulting in the only remains left of a decayed and fragmented cartilaginous fish. Crownward chondrichthyans furthermore grow, shed and replace their teeth throughout their lifetime which may further complicate this bias [99,100]. Rates of replacement in fossil taxa are mostly unknown and suggested to have been slow [101] and there is good evidence of tooth retention throughout life in Palaeozoic cladodonts [99].

Isolated teeth and fin spines still contain important information on the taphonomic filters of preservation as well as temporal and spatial distribution and should not be strictly excluded [67]. Furthermore, the overall patterns of peaks and drops in chondrichthyan completeness are mostly retained between the original data and the subsets excluding isolated remains, which indicates that the same trends in completeness over time are recovered from all datasets even though absolute values of completeness change considerably (figure 2). Without the acanthodian fossils, completeness scores throughout the Silurian are limited to putative chondrichthyan taxa such as *Kannathalepis*, *Frigorilepis* and *Wellingtonella* [102–104] as well as isolated scale-based taxa from elegestolepidid and mongolepidid chondrichthyans and isolated fin spine-based taxa from the order Sinacanthida [73,74,105,106]. The addition of the acanthodian completeness scores to the chondrichthyan dataset significantly increases absolute values of completeness for the Silurian and Lower to Middle Devonian, but has little effect on completeness scores post-Devonian (figure 2). These findings reflect the new phylogenetic consensus that recognizes acanthodians as part of the chondrichthyan stem-group [10,17,26–29,107–112], bridging a *ca* 30 Myr gap in their fossil record.

### 4.1. Comparisons with other vertebrate groups

The acanthodian data from Schnetz *et al*. [68] represent the only other available data on Palaeozoic and fish group skeletal completeness, and both acanthodians and non-acanthodian chondrichthyans show very similar completeness patterns. Acanthodians and traditionally recognized chondrichthyans overlapped throughout the Palaeozoic era, are found in the same formations and localities, and even show evidence of direct interactions [113]. This is hardly surprising given that the distinction between the groups is arbitrary, and blurred by genera such as *Doliodus.* Similarities in preservation should therefore be expected: anatomical constructions are fundamentally similar. Informally, it was often suggested that the main difference between a Devonian acanthodian and shark was size, acanthodians being characterized as mostly around 20 cm or less in length [114]. Comparison of completeness with other temporally and anatomically similar groups such as Palaeozoic actinopterygians are needed to provide a worthwhile, albeit large and challenging, dataset to compare groups from similar ecological niches.

Given the lack of available fish completeness data, comparisons with other marine vertebrate groups may shed some light on the fundamental processes underlying observed completeness patterns. Initial chondrichthyan completeness is considerably lower than completeness of other marine vertebrate groups such as Mesozoic plesiosaurs and ichthyosaurs, which exhibit the highest overall and median skeletal completeness records of all published tetrapod groups [65,66] (figure 4). However, chondrichthyan completeness scores increase substantially upon exclusion of isolated skeletal remains such as isolated teeth, scales and fin spines, rising higher than values calculated for most terrestrial tetrapod groups, including sauropodomorphs, theropods and pelycosaurs, a pattern also shown in acanthodians. However, it is worth noting that many taxa within groups such as theropods are also based on highly fragmentary material (e.g. teeth for theropod dinosaurs; vertebrae for sauropodomorph dinosaurs). While the median SCM2 values of chondrichthyans and acanthodians are still somewhat lower than the completeness values of plesiosaurs and ichthyosaurs, our results strengthen the argument that different taphonomic processes are at play in the vertebrate records between marine and terrestrial environments [66,115]. Early chondrichthyans, including acanthodian chondrichthyans, are considerably geologically older than any of the tetrapod groups for which completeness patterns have been published and might therefore face additional difficulties (i.e. filters) in terms of the preservation of their preservational history. Likewise, the differences in skeletal composition between chondrichthyans and tetrapods probably affect their patterns of completeness. While comparisons between both temporally and taxonomically distinct groups may seem strange at first glance, it allows us to highlight differences and similarities of fossil record completeness patterns of different vertebrate groups in the absence of such data for similar Palaeozoic groups.

### 4.2. Completeness variation between chondrichthyan taxonomic groups

Analyses of the SCM2 values for the chondrichthyan subclasses reveal highly similar completeness patterns between phylogenetic divisions into Elasmobranchii and Holocephali and traditional taxonomic schemes such as Elasmobranchii and Euchondrocephali. Median completeness values for each of these are very low, indicating a majority of isolated fragments, mainly isolated teeth, in each of the groups. The elasmobranch record is more complete than the holocephalan/euchondrocephalan record, albeit only by around 0.55% with regards to median values. Grouping into Palaeozoic chondrichthyan orders further amplifies the pattern of a high number of species recovered from fragmented and isolated remains, with the occasional discovery of a highly complete species, in most of the groups (electronic supplementary material, figure S3). In some of the investigated groups, the completeness distributions are extreme and consist of identical SCM2 values for each taxon included in the group, thus showing no further distribution than the median. This means that these groups are known entirely from specific isolated material, predominantly isolated teeth, isolated scales or isolated fin spines, e.g. Bransonelliformes, Mongolepidida and Sinacanthida.

By contrast, some of the groups show noticeably different SCM2 distribution patterns: the chimaera-like Iniopterygia have the highest median completeness of any group. The significantly higher completeness of iniopterygians is most likely the result of a combination of contributing factors: they are limited to 10 species and two taxa indeterminate at genus and species level and are almost entirely known from partial and complete skeletons, with findings of isolated skeletal remains being the exception. Additionally, iniopterygians are temporally and geographically constrained. They have been exclusively recovered from shale settings, which strikingly differ in terms of environment, belonging to either the very shallow nearshore waters in the Mecca Quarry Shale and Bear Gulch Limestone [116–118], or the deep offshore deposits of the Stark and Wea Shales [116,119], indicating potential environmental influences on preservation. Three-dimensional nodules with iniopterygians have additionally been found from the boundary between the Haskell Limestone Member and the overlying Robbins Shale of the Stranger Formation [120]. The Antarctilamniformes comprise a small group of Devonian chondrichthyans whose fossil discoveries, including braincase, fin spine and tooth elements, have added important insights into the record of earliest chondrichthyans [121–124] which is also reflected in their higher median and interquartile SCM2 distribution pattern compared to the majority of chondrichthyan orders. The Symmoriiformes show a range of both low and high completeness value distributions. This is not surprising given the prominent members of this group such as *Symmorium* [125], *Akmonistion* [95], *Cobelodus* [126] or *Falcatus* [127] which are all known from at least partially articulated skeletons, while the low completeness levels stem from species described from isolated remains alone such as *Kungurodus* [128] or *Denaea williamsi* [129].

### 4.3. Geographical controls on chondrichthyan completeness

Chondrichthyan fossils are recovered from all continents but vary greatly in terms of abundance. While there are no recovered significant differences between chondrichthyan completeness from the modern Northern and Southern Hemisphere localities, total species numbers differ considerably (figure 5*a*). Chondrichthyan richness is dominated by species from North America and Europe. While this could be indicative of a higher diversity of chondrichthyans within these regions, it is more likely to represent a bias of greater collection effort, and a historical trend of greater sampling and outcrop availability within these areas, as has been shown for the temporally and anatomically similar actinopterygians [34]. Sampling of any given fossil record varies geographically and across stratigraphic units introducing spatial biases resulting from underlying geological and anthropogenic factors [53]. In addition, the effects of climate, vegetation cover and erosion can further restrict the exposure of otherwise consistently fossiliferous horizons [61]. Our findings highlight the potential of a wealth of undiscovered information on the chondrichthyan fossil record in the southern regions that could be crucial in terms of expanding our knowledge on chondrichthyan diversity and evolution.

There is no significant geographical variation in chondrichthyan SCM2 values, meaning that the considerably smaller number of taxa described from the Southern Hemisphere and continents (including Africa, South America, Australia and Oceania and Antarctica) contain similar mean completeness levels to the taxonomically richer records from the Northern Hemisphere continents (North America, Europe and Asia; figure 5). North America, Europe and Asia do however show the highest range of completeness values compared to the southern continents, signifying that the individually most complete chondrichthyan taxa are found there even though this is not reflected in the mean levels of completeness. The chondrichthyan fossil record of South America exhibits the overall lowest SCM2 distribution and variability in the Palaeozoic which coincides with previous suggestions regarding its scarce and discontinuous nature of the Devonian vertebrate record [122,130,131]. In accordance with this, South American acanthodians show a similarly limited fossil evidence of both skeleton completeness as well as taxonomic abundance [68]. South America's fauna is suggested to be dominated by chondrichthyans and acanthodians during Devonian times, but these observations are based on assemblages of mostly fragmented material, including fin spines, endoskeletal elements and scales [131]. This pattern is not only limited to the Devonian but seemingly ranges throughout the Palaeozoic. Again, we suspect that lack of research attention (sampling bias) is a major contributory factor.

### 4.4. Palaeoenvironmental and palaeoecological biases

The significant inverse relationship evident between acanthodian completeness and sea level [68] recurs when the dataset is expanded to include the chondrichthyan total group (figure 6 and table 1). This relationship may be surprising given that higher sea levels can place bottom waters and basins in deep shelf areas below physical barriers resulting in anoxic conditions [132,133]. These conditions together with lower energy deposition and less scavenging and weathering in deeper water environments are considered to provide better conditions for preservation than shallow waters [67,134]. However, besides chondrichthyans and acanthodians, inverse relationships between sea level and completeness have also been shown in the marine ichthyosaurs [65], plesiosaurs [66] and the decidedly terrestrial sauropodomorphs [52,63]. Curiously, this correlation is absent in the marine mosasaur record [67]. Osteostracans and non-psammosteid heterostracans, early jawless fishes, also show an inverse proportional relationship between recovery potential of fossils and sea level; however, this probably reflects their restriction to shallow-water environments [135].

Contrary to acanthodians [68], non-acanthodian chondrichthyan skeletal completeness does not show a strong trend towards higher values from either freshwater or marine environments in the Palaeozoic. This absence of trend may be amplified by the high amount of chondrichthyan fossils found from both BA0 and BA1/BA1–2 environments. BA0, BA1 and BA2 zones exhibit similar patterns of completeness throughout the Palaeozoic. This may relate to a considerable number of anadromous taxa, which migrate between freshwater and marine environments, inhabiting the aquatic environments in the Palaeozoic. BA0 and BA1 SCM2 values are not significantly different from each other which further suggests the dominance of anadromous chondrichthyans in the dataset. The close relationship between the marine BA1 and BA2 zones is not surprising as intertidal and shallow subtidal environments are often coupled in interpretations of lithostratigraphic units (see the electronic supplementary material dataset) and there may be a limitation in resolution when it comes to differentiating between the two very similar environments [68].

Chondrichthyan skeletal completeness sorted by BAs further reveals higher values in specific BAs at different points throughout the Palaeozoic which indicates potential ecological (habitat preferences) or preservational (depositional environment) biases. Chondrichthyan SCM2 is initially restricted to intertidal and shallow subtidal marine environments (BA1–BA2) in the Ordovician and Silurian and freshwater SCM2 is only found from the Devonian onwards. Chondrichthyan fossils are found in BA3 and BA4 (middle to outer shelf, reef settings and subtidal dynamic environments) from the earliest Silurian through to the end of the Permian but show a generally low completeness except for spikes in the Late Mississippian and Late Pennsylvanian (figure 7). A similar pattern has been recovered in acanthodians and attributed to a combination of environmental deposition and sampling bias [68]. Indeed, the vast majority of chondrichthyan records from this interval and environment are derived from one stratigraphic unit, the North American Heath Formation Lagerstätte, where individuals were rapidly buried in a wide shallow freshwater-influenced lagoon, preventing the warm water setting to rapidly decay and disarticulate the fish bodies [118,136]. Thus, the spike found in BA3 and BA4 from the Late Mississippian most likely accounts for a sampling bias rather than a genuine signal for high completeness in these BAs.

Extension of SCM2 values into truly deeper water settings (BA5 and BA6) only occur from the Middle Devonian on. This may well coincide with an expansion into more diverse habitats and an availability of niches following the Hangenberg extinction at the Devonian–Carboniferous boundary as has been suggested before [137]. Interestingly, in the latest stages of the Permian prior to the end-Permian mass extinction, SCM2 is high in both BA0 and BA5–6 even though few specimens are recovered, and shows that chondrichthyans may still occupy a high variety of habitats towards the end of the Palaeozoic. Acanthodian fossils are generally absent from deep water settings while highly complete chondrichthyans are recovered from deep water marine environments (BA5 and BA6) in the Late Devonian, throughout most of the Carboniferous and the later Permian stages (figure 7). The significant correlations between the deep-water BAs (BA5 and BA6) with total SCM2 further illustrate the significant role they play in chondrichthyan completeness (table 2). BA0, which accounts for freshwater settings, conversely, does not significantly correlate with total completeness and may provide less of an explanation for the freshwater versus marine trends in chondrichthyan completeness.

The relationship between sea level, environment and skeletal completeness warrants further investigation. At the level of our analyses, the relationship with sea level through the Palaeozoic seems to correspond to the first-order variation probably influenced by the WIlson Cycle (repeated opening and closing of ocean basins; e.g. [138,139]). However, understanding what the response is at a more granular level (within basin and within sequence) may yield further insights following a stratigraphic palaeobiological approach (*sensu* [140,141]). Unfortunately, the published literature and data from museum collections currently lacks sufficient resolution to place skeletal completeness of chondrichthyans in such sequence stratigraphic framework.

## 5. Conclusion

The Palaeozoic chondrichthyan fossil record is subjected to spatial, temporal and environmental biases, and is dominated by isolated teeth, scales and fin spines, which obscure much of the patterns and trends of completeness through the Palaeozoic. Modern Northern Hemisphere localities do not yield significantly more complete chondrichthyans than the southern counterpart, albeit having a significantly higher total number of species, indicating a potential wealth of undiscovered information in the global south. Higher completeness is reported in specific environmental zones at different points throughout the Palaeozoic and correlates negatively with sea level, indicating potential ecological and preservational biases. Chondrichthyans exhibit the poorest completeness range of any of the previously investigated vertebrate groups but data from suitably comparable groups, such as Actinopterygii, are missing. The absence of a consistent application of a taxonomic concept for naming and assigning chondrichthyan taxa based on isolated scales and fin spines, coupled with a potential for taxonomic ‘oversplitting', probably explains at least some of the observed low completeness trends. The influences of these biases illustrate the challenges faced when attempting to estimate chondrichthyan macroevolutionary patterns, based upon what is quantified here as a spatially, temporally and taxonomically incomplete fossil record.

## Data Availability

The data are provided in the electronic supplementary material [142].
